# Regulation of intraocular pressure by microRNA cluster miR-143/145

**DOI:** 10.1038/s41598-017-01003-z

**Published:** 2017-04-19

**Authors:** Xinyu Li, Fangkun Zhao, Mei Xin, Guorong Li, Coralia Luna, Guigang Li, Qinbo Zhou, Yuguang He, Bo Yu, Eric Olson, Pedro Gonzalez, Shusheng Wang

**Affiliations:** 1 Department of Ophthalmology, Tongji Hospital, Tongji Medical College, Huazhong University of Science and Technology, 1095 Jiefang Road, Wuhan, Hubei 430030 P.R. China; 2 Department of Ophthalmology, the Fourth Affiliated Hospital of China Medical University, Eye Hospital of China Medical University, Key Lens Research Laboratory of Liaoning Province, Shenyang City, Liaoning Province, China; 3 Department of Cell and Molecular Biology, Tulane University, 2000 Percival Stern Hall, 6400 Freret Street, New Orleans, LA 70118 USA; 4 Department of Ophthalmology, Tulane University School of Medicine, 1430 Tulane Avenue, SL-69, New Orleans, LA 70112 USA; 5 Department of Ophthalmology, University of Texas Southwestern Medical Center, 5323 Harry Hines Boulevard, Dallas, TX 75390 USA; 6 Cincinnati Children’s Hospital Medical Center, Department of Pediatrics, University of Cincinnati, Cincinnati, 45247 OH USA; 7 Department of Molecular Biology, University of Texas Southwestern Medical Center, 5323 Harry Hines Boulevard, Dallas, TX 75390 USA; 8 Department of Ophthalmology, Duke University, Durham, North Carolina USA

## Abstract

Glaucoma is a major cause of irreversible blindness worldwide. Elevated intraocular pressure (IOP), which causes optic nerve damage and retinal ganglion cell death, is the primary risk factor for blindness in glaucoma patients. IOP is controlled by the balance between aqueous humor secretion from the ciliary body (CB) and its drainage through the trabecular meshwork (TM). How microRNAs (miRs) regulate IOP and glaucoma *in vivo* is largely unknown. Here we show that miR-143 and miR-145 expression is enriched in the smooth muscle and trabecular meshwork in the eye. Targeted deletion of *miR-143*/*145* in mice results in significantly reduced IOP, consistent with an ~2-fold increase in outflow facilities. However, aqueous humor production in the same mice appears to be normal based on a microbeads-induced glaucoma model. Mechanistically, we found that miR-143/145 regulates actin dynamics and the contractility of TM cells, consistent with its regulation of actin-related protein complex (ARPC) subunit 2, 3, and 5, as well as myosin light chain kinase (MLCK) in these cells. Our data establish miR-143/145 as important regulators of IOP, which may have important therapeutic implications in glaucoma.

## Introduction

Glaucoma is the leading cause of irreversible vision loss, affecting ~70 million people worldwide^1^. Although the pathogenesis of glaucoma remains unclear, it is defined as progressive degenerative disease characterized by the death of the retinal ganglion cells (RGC)^2^. Elevated intraocular pressure (IOP) is closely related to RGC death, and represents the primary risk factor for blindness in glaucoma patients. Reducing IOP is the only proven method to treat glaucoma to date^3^. The current IOP-reducing drugs include prostaglandin analogues, β-adrenergic blockers, α-adrenergic agonists, carbonic anhydrase inhibitors, and cholinergic agonists. For the majority of patients, IOP management in glaucoma normally involves more than a single class of drugs. Therefore, finding underutilized therapeutic targets is necessary to push the IOP-lowering effect of drugs beyond the current levels.

IOP is determined by the balance between aqueous humor secretion from the ciliary body and its drainage through the trabecular meshwork (TM) and uveoscleral outflow pathways. The genetic mechanisms of IOP regulation are only partially understood. For example, mutations in *MYOC*, the gene encoding myocilin, result in decreased outflow and elevated IOP, but account for only 4% of the glaucoma cases^4^. The trabecular outflow pathways are being heavily investigated as therapeutic targets for glaucoma. Trabecular outflow is driven by the pressure differential between the interior (IOP) and the exterior (episcleral venous pressure) of the eye^5^. In the outflow pathway, the aqueous humor filters through the TM, and reaches the juxtacanalicular (JCT) region near the inner wall of Schlemm’ canal (SC). The funneling mechanism of the inner wall creates a bottleneck that generates resistance and IOP. At the cellular and molecular level, IOP reduction can be achieved by increasing trabecular outflow through decreasing the volume of cells that populate the JCT region, decreasing the contractility or increasing the relaxation properties of TM cells, decreasing the amount and type of extracellular matrix (ECM), or improving the funneling mechanism of the JCT. Drugs that target TM contraction or relaxation, including sphingosine-1-phosphate 2 (S1P2) receptor antagonists that block myosin light chain (MLC) phosphorylation, and Rho kinase or LIM-kinase 2 inhibitors, have been shown to relax TM cells, increase outflow facilities, and lower IOP^6–9^.

microRNAs (miRNAs or miRs) are small noncoding RNAs that repress multiple target genes and are implicated in numerous ophthalmic pathologies^10^. Several studies have established the involvement of miRNAs in glaucoma. miR-29 family genes have been shown to regulate the expression of multiple ECM components in TM cells^11–13^. miR-24 regulates the induction of TGFβ1 in TM cells in response to cyclic mechanical stress^14^. miR-200c inhibits the contraction and reduces traction force in TM cells, and miR-200c mimic has been shown to decrease IOP by ~25% in rats^15^. Conversely, inhibition of miR-200c using an adenoviral vector expressing a molecular sponge to miR-200c led to a significant increase in IOP. These results establish the potential of modulating trabecular contraction and IOP by miRNA *in vivo*. However, genetic evidence of miRNA involvement in IOP regulation is still lacking. Here we show that the miR-143/145 cluster is expressed in the smooth muscle and TM cells in the eye. Deletion of *miR-143* and *miR-145* in mice results in an ~19% decrease in IOP, which is consistent with an ~2-fold increase in outflow facilities. Mechanistically, miR-143/145 regulates actin dynamics and TM cell contractility, consistent with its regulation of actin-related protein complex (ARPC) subunit 2, 3, and 5, as well as myosin light chain kinase (MLCK) in these cells. Our results demonstrate that miR-143/145 regulates IOP and outflow facilities *in vivo*, suggesting that manipulating miR-143/145 level may have therapeutic implications in glaucoma.

## Results

### Expression of miR-143 and miR-145 in smooth muscle and TM cells in the eye

**Figure 1:**
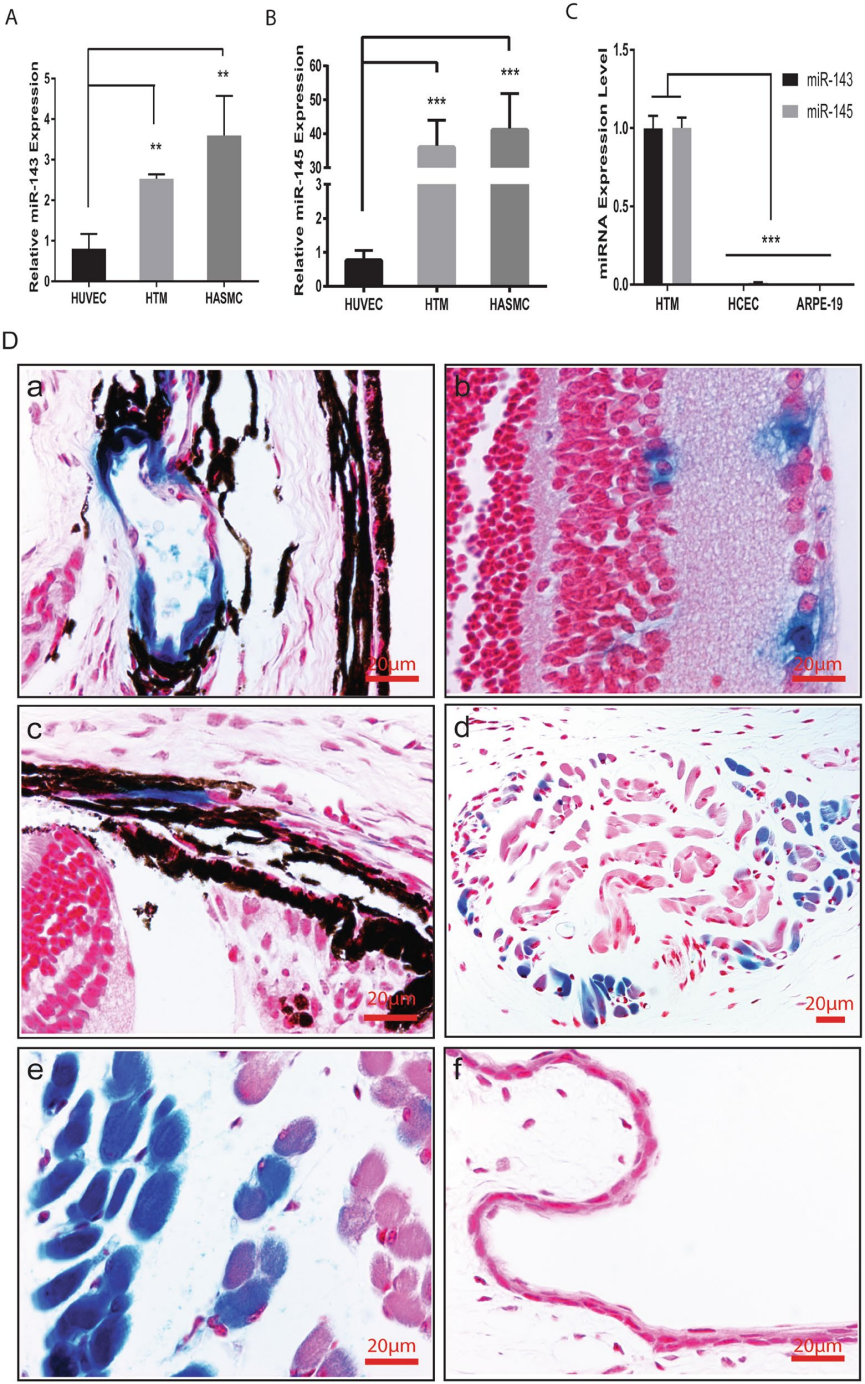
Expression of miR-143 and miR-145 in the eye as revealed by qRT-PCR and LacZ staining. LacZ plus eosin counter-staining was performed in ~2-month old transgenic mice in which LacZ is driven by a 5 kb miR-143/145 enhancer. (**A**) Relative expression of miR-143 in HTM cells compared to HUVEC and HASMC cells. **p < 0.01; (**B**) Relative expression of miR-145 in HTM cells compared to HUVEC and HASMC cells. ***p < 0.001; (**C**) Relative expression of miR-143/145 in HTM cells compared to HCEC and ARPE-19 cells. ***p < 0.001; (**D**) LacZ staining was observed in smooth muscle (a), pericytes (b), ciliary muscle (c), extraocular muscle (d,e), but not in the corneal epithelial layer (f) in the eye.

The miR-143/145 cluster has been shown to be enriched in vascular smooth muscle cells^16–19^. Deletion of miR-143 and miR-145 results in a significant reduction (~14% decrease) in systolic blood pressure due to reduced vascular tone^19^. To establish a potential role of miR-143/145 in IOP regulation, we first examined their expression in TM cells and compared them with that in SMC cells. Human TM (HTM) cells were isolated from donor eyes with no history of eye disease, and the expression of miR-143 and miR-145 was examined by qRT-PCR. miR-143/145 expression in HTM cells appeared to be less but comparable to the expression in human aortic smooth muscle cells (HASMC), but more abundant than in human umbilical vein endothelial cells (HUVEC) (Fig. 1A,B). When different ocular cell types were compared, miR-143/145 expression in HTM cells was ~100–1000 times higher than that in human ARPE-19 or human choroidal endothelial cells (HCEC), confirming the SMC and TM enrichment of miR-143/145 expression (Fig. 1C). To further determine whether a 5.5 kb miR-143/145 upstream regulatory sequence drives miR-143/145 expression in the eye, beta-galactosidase (LacZ) staining was performed in transgenic mice in which a 5.5 kb of genomic DNA upstream of miR-143/145 was fused to a lacZ reporter^16^. Robust transgene expression was observed in the vascular SMCs, pericytes (PCs), and ciliary muscles (CMs) of the eye (Fig. 1D). The identity of these cell types was confirmed by immunostaining with α-SMC (a smooth muscle marker) and NG2 (a pericyte marker) antibodies (Supplemental Fig. 1). In addition, LacZ reporter activity was also observed in the extraocular muscles (Fig. 1D). miR-143 and miR-145 have been reported to be expressed in corneal epithelial cells, especially limbal stem cells^20, 21^. However, miR-143/145 expression level was not detectable in corneal or retinal epithelium in the LacZ reporter mice (Fig. 1D). The reporter activity was not detectable in the TM of the reporter mice, although miR-143 and miR-145 was detected in HTM cells by qRT-PCR. Based on these data, we conclude that miR-143 and miR-145 are enriched in the SMCs, pericytes, CMs and extraocular muscle in the mouse eye, but are also expressed in the TM cells.

### Normal gross ocular morphology in *miR-143*/*145* dKO mice

**Figure 2:**
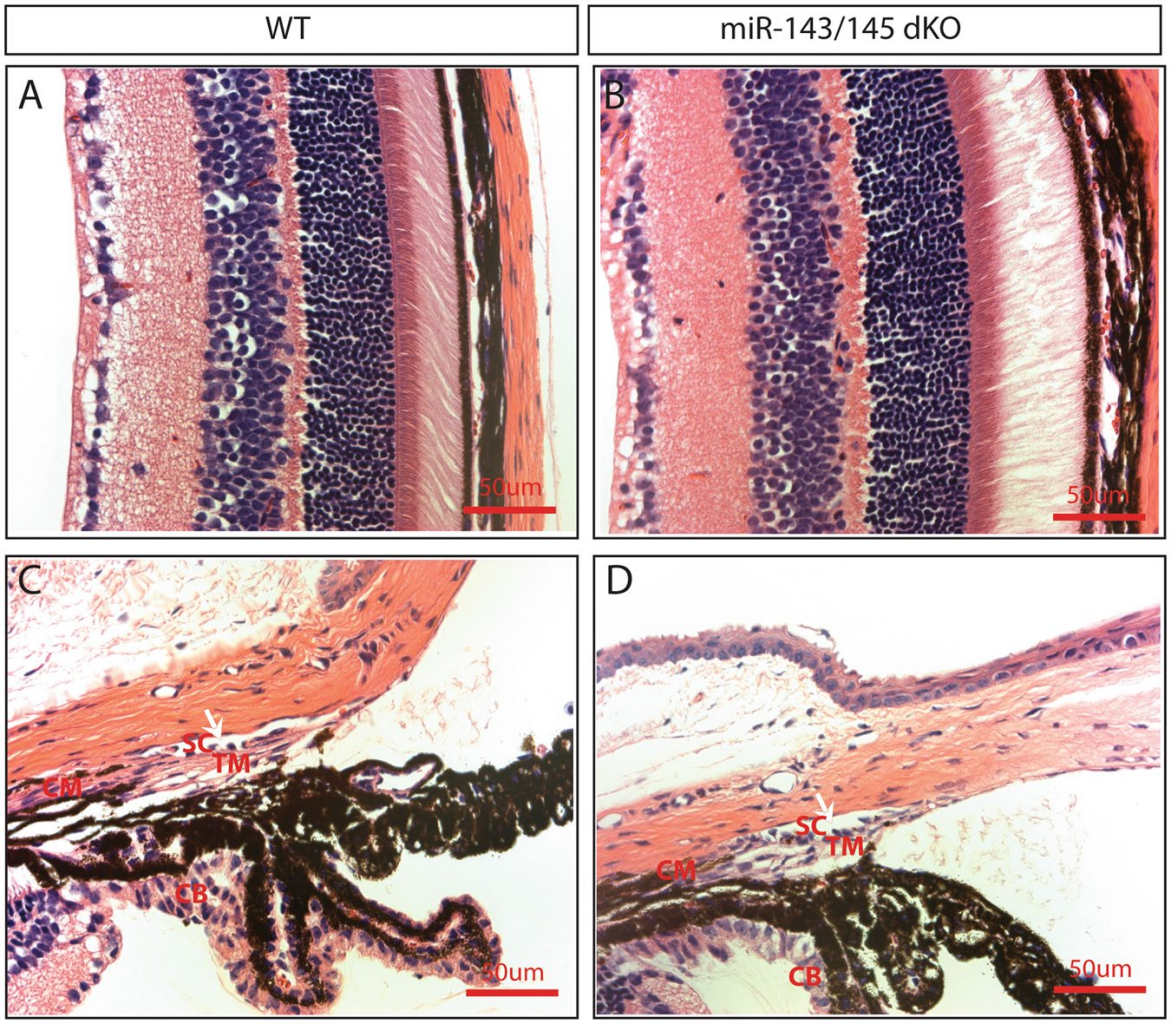
Normal histology in the eyes of *miR-143*/*145* dKO mice. No obvious difference was observed in the retinal structure (**A**,**B**), ciliary muscle (CM), trabecular meshwork (TM), ciliary body (CB), and the area of Schlemm’s canal (SC) in ~2-month old *miR-143*/*145* dKO (**D**) mice compared to that in wildtype control littermate mice (**C**) by H&E staining.

Encouraged by the smooth muscle- and TM-enriched expression of miR-143 and miR-145 in the eye, we set to characterize the ocular phenotype in the *miR-143*/*145* double knockout (dKO) mice that we had previously generated^16^. These mice are viable without overt gross abnormalities^16–19^. Hematoxylin and Eosin staining was used to study the baseline phenotype in the dKO mice. No difference was found in the retinal layers in *miR-143*/*145* dKO mice compared to wild-type (WT) littermate controls (Fig. 2A,B). Gross morphology and the ultrastructure of extraocular muscles also appeared normal in the dKO mice (Supplemental Fig. 2). Iridocorneal angle structure is critical for aqueous humor outflow pathway and IOP regulation. No obvious differences were observed in the iridocorneal structures (Fig. 2C,D). The structural integrity of TM, CM, cilary body (CB), as well as the area of Schlemm’s canal (SC), appeared normal in *miR-143*/*145* dKO mice compared to the controls. Together, these results suggest that miR-143/145 is not required for the development of iridocorneal and retinal structures.

### Reduced IOP in *miR-143*/*145* dKO mice

**Figure 3:**
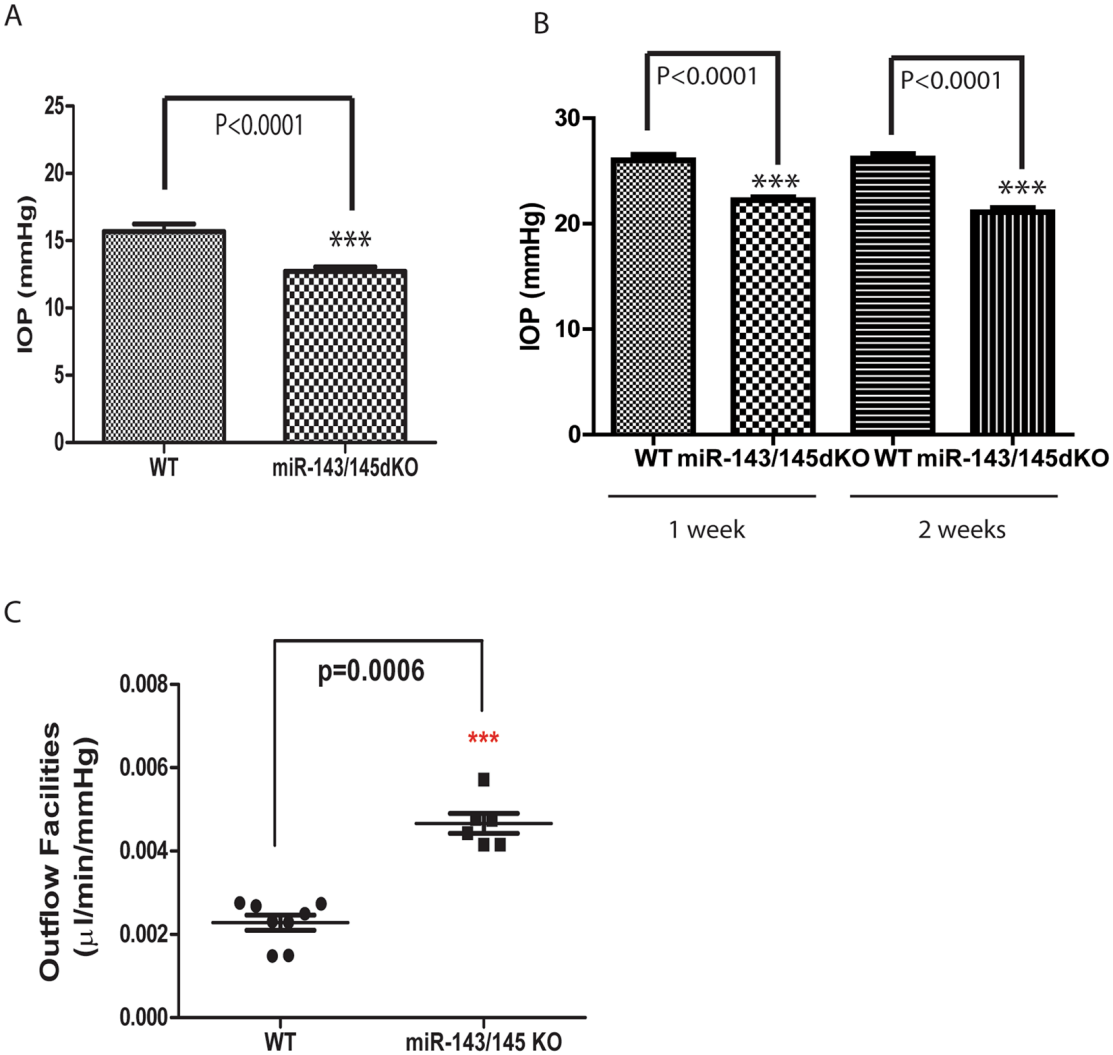
Reduced IOP and increased outflow facilities in *miR-143*/*145* dKO mice. (**A**) Reduced intraocular pressure (IOP) in *miR-143*/*145* dKO mice. ***p < 0.0001; (**B**) Reduced intraocular pressure in *miR-143*/*145* dKO mice in an experimental glaucoma model. The IOP of the mice was measured at 1 and 2 weeks after microbeads injection into the eye. ***p < 0.0001; (**C**) Significant increase in outflow facilities in 16-month-old *miR-143*/*145* dKO mice compared to that in WT control mice. ***p < 0.0006.

To test whether miR-143 and miR-145 are required for regulating IOP in mice, we measured the day-time IOP in *miR-143*/*145* dKO mice and WT littermate controls using tonometry. These measurements were performed in anesthetized adult mice. While the average IOP in the WT mice was ~15.7 mmHg (15.7 ± 0.6 mmHg, N = 23), the IOP in *miR-143*/*145* dKO mice was only ~12.7 mmHg (12.7 ± 0.3 mmHg, N = 24) (Fig. 3A). Therefore, the IOP in *miR-143*/*145* dKO mice was ~19% less than that in the WT littermates (P < 0.0001).

To further examine whether miR-143 and miR-145 are required for regulating IOP under high IOP conditions, we induced intraocular hypertension in these mice by polystyrene microbead injection, and examined the effects of *miR-143*/*145* deletion on IOP elevation^22, 23^. Microbeads obstruct the aqueous humor outflow, and can be used to determine whether the aqueous humor production or aqueous humor outflow is affected in *miR-143*/*145* dKO mice. In WT mice, the IOP increased from ~15.7 mmHg to ~26.2 mmHg (26.2 ± 0.4, N = 12) at 1 week and to ~26.0 mmHg at 2 weeks after injection of microbeads into the anterior chamber of mice. In miR-143/145 dKO mice, the IOP increased from ~12.7 mmHg to ~22.2 mmHg (22.2 ± 0.3, N = 12) at 1 week and to ~21.1 mmHg (21.1 ± 0.4, N = 12) at 2 weeks after microbeadinjection (Fig. 3B). Although the IOP in *miR-143*/*145* dKO mice was still significantly less than that in the WT littermates at 1 or 2 weeks after microbead injection (P < 0.0001), the IOP elevation was comparable (10.5 and 10.3 mmHg increase in WT vs 9.5 and 8.4 mmHg in dKO at 1 and 2 weeks post injection, respectively) in both mice after microbead injection. These results suggest that the aqueous humor production is largely normal but the outflow facilities are increased by *miR-143*/*145* knockout, which results in the decreased IOP in those mice.

To determine whether the outflow facilities were increased in *miR-143*/*145* dKO mice, 8 WT and 6 littermate dKO mice eyes were perfused, and the outflow facilities were measured at pressures of 15, 25, and 35 mm Hg, respectively^24^. Outflow facilities were twice as high in dKO mice than in WT controls (0.0023+/−0.00018 µl/ml/mmHg in WT (N = 8) v.s. 0.0047+/−0.00023 µl/ml/mmHg in dKO mice (N = 6), p < 0.0006), confirming our prediction from the microbead experiments (Fig. 3C). These data demonstrate that the decreased IOP in the *miR-143*/*145* dKO mice results from a ~2-fold elevation in the outflow facilities in these mice.

### miR-143 and miR-145 regulate actin dynamics and contractility of HTM cells

**Figure 4:**
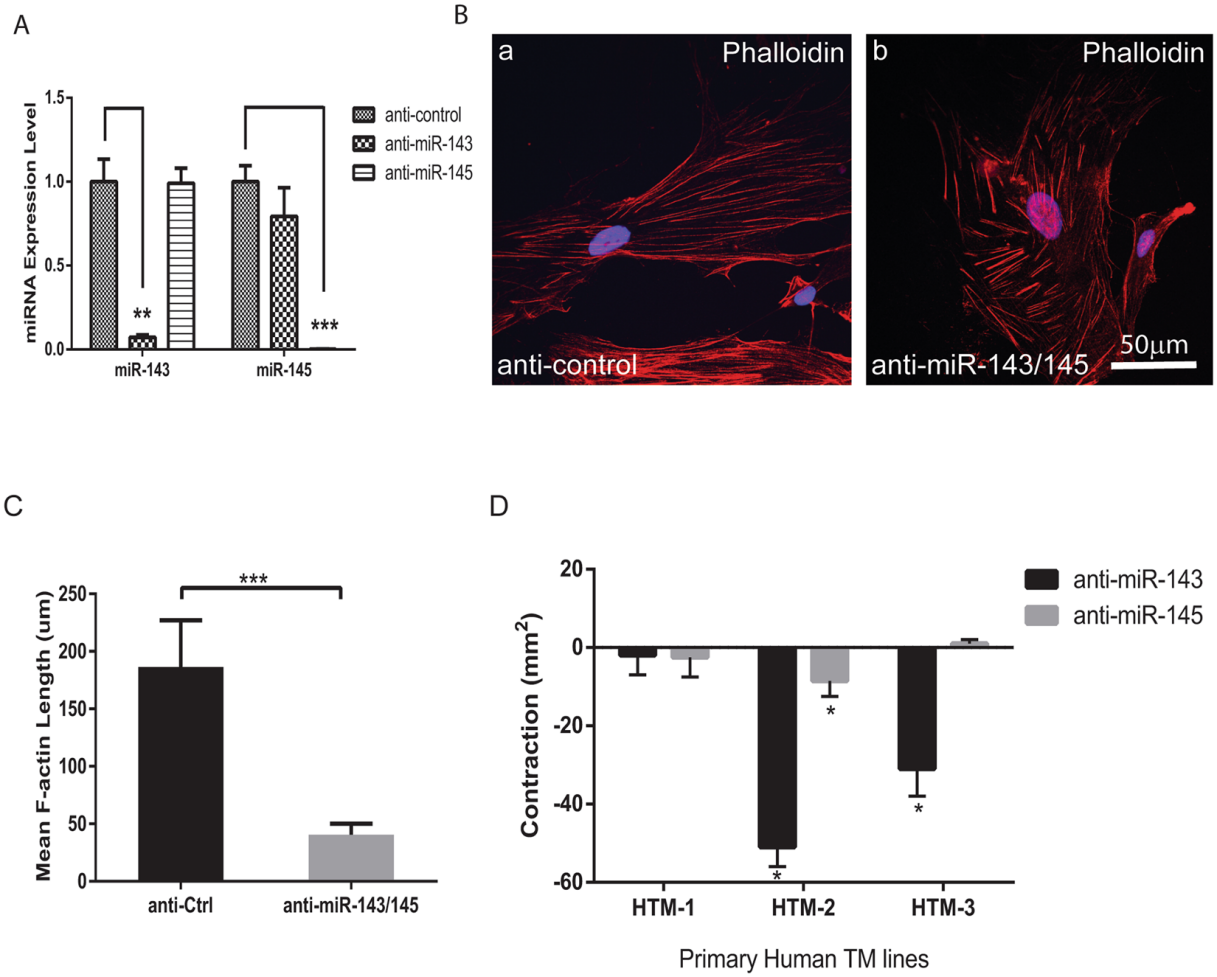
Effect of miR-143/145 silencing on stress fiber length and cell contractility in HTM cells. (**A**) Efficient knockdown of miR-143 or miR-145 by respective antagomiR in HTM cells. **p < 0.01; ***p < 0.001; (**B**) Stress fibers stained by phalloidin in HTM cells transfected with miR-143 and miR-145 antagomiR compared to a control antagomiR. Scale = 50 µm; (**C**) Quantification of stress fiber length (µm) in (**B**) from an average of 6–7 fields per group. ***p < 0.001; (**D**) Effect of miR-143/145 silencing on HTM cell contractility. Basal level of contraction for each of the three cell lines analyzed was calculated as the difference in area between cells transfected with antagomiRs to control, miR-143 or 145. Bars represent standard deviation in three independent experiments for each cell line. *p < 0.05.

To further dissect the mechanism whereby miR-143 and miR-145 regulate outflow facilities, we tested the effect of miR-143/145 silencing on the actin dynamics and contractility of TM cells. miR-143 and miR-145 have been shown to regulate contractility and maintain actin stress fibers in smooth muscle cells^16–19^. Their function in TM cells is still unknown. miR-143 and miR-145 antagomiRs were used to silence these two miRNAs, respectively. More than 90% knockdown of miR-143/145 in HTM cells was achieved as shown by qRT-PCR (Fig. 4A). miR-143 antagomiR only specifically silenced miR-143 expression, without affecting expression of miR-145; and vice versa. To visualize the actin stress fibers in miR-143/145 silenced HTM cells, the amount and distribution of filamentous F-actin and the number of stress fibers were visualized by labeling with phalloidin (Fig. 4B,C). Abundant long F-actin labeled stress fibers were present in the control cells. Stress fiber length was significantly reduced in HTM cells transfected with miR-143 and miR-145 antagomiRs (p < 0.001). We also examined whether miR-143/145 silencing affects HTM cell contractility. Transfection of HTM cells with miR-143 antagomiR resulted in a significant decrease in cell contraction in two of the three primary HTM cell lines analyzed (p < 0.0018) and had no significant effect in the other cell line (p < 0.3) (Fig. 4D). The antagomir for miR-145 resulted in only a moderate but significant inhibition of cell contraction in one of the cell lines (p < 0.023). These results suggest that miR-143/145 regulates the contractility of HTM cells, with miR-143 having a more dominant role than miR-145.

### Regulation of multiple genes involved in actin dynamics and contractility by miR-143/145 in HTM cells

**Figure 5:**
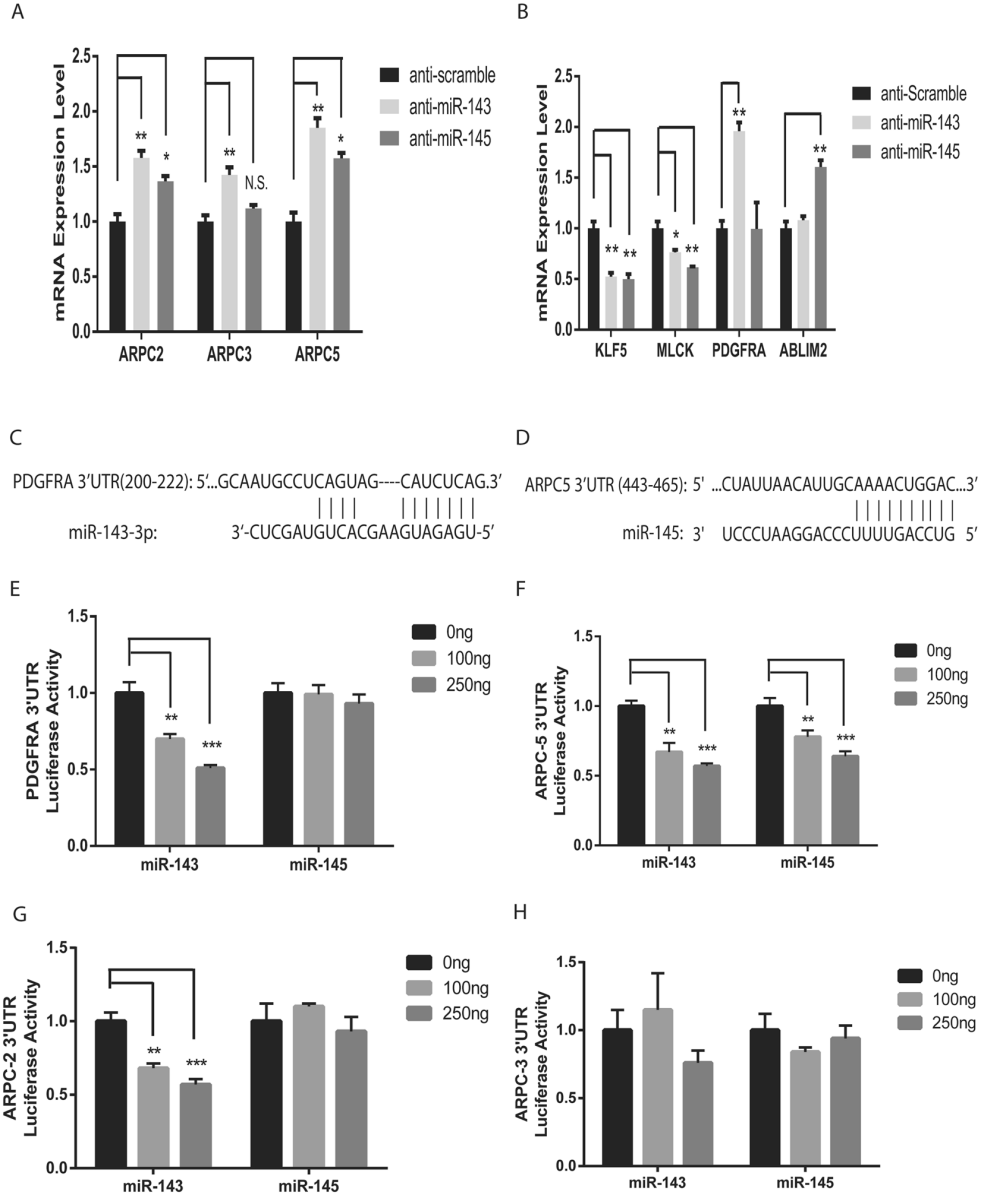
Regulation of miR-143/145 target genes and glaucoma-related genes by miR-143/145. (**A**) Upregulation of ARPC-2, -3 and -5 by miR-143 or miR-145 silencing. *p < 0.05, **p < 0.01, N.S., not significant; (**B**) Downregulation of KLF5 and MLCK by miR-143 or miR-145 silencing. PDGFRA was upregulated by miR-143 silencing, and ABLIM2 was upregulated by miR-145 silencing. *p < 0.05, **p < 0.01; (**C**) Sequence alignment of PDGFRA 3′-UTR and miR-143-3p, with complementary nucleotides indicated by lines; (**D**) Sequence alignment of ARPC5 3′UTR and miR-145-5p, with complementary nucleotides indicated by lines; (**E**) miR-143 but not miR-145 overexpression repressed PDGFRA 3′-UTR activity in a dose-dependent manner. **p < 0.01, ***p < 0.001; (**F**) Either miR-143 or miR-145 overexpression repressed ARPC5 3′UTR activity in a dose-dependent manner. **p < 0.01; ***p < 0.001. (**G**) miR-143 but not miR-145 overexpression repressed ARPC-2 3′-UTR activity in a dose-dependent manner. **p < 0.01, ***p < 0.001; (**H**) Neither miR-143 nor miR-145 overexpression repressed ARPC-3 3′-UTR activity.

Although not homologous, miR-143 and miR-145 share a number of common target genes involved in actin dynamics and contractile function^16, 18, 19^. To identify miR-143/145 target genes and regulated genes that contribute to the reduced cell contraction and actin filaments in miR-143/145 silenced HTM cells, the expression of a list of predicted and/validated miR-143/145 target/regulated genes was examined by qRT-PCR. The selected genes, including ARPC-2, ARPC-3, ARPC-5, kruppel-like factor (KLF) 4, KLF5, rock kinase (ROCK) 1, actin-binding LIM domain protein (ABLIM) 2, platelet-derived growth factor receptor (PDGFR) A, protein kinase C epsilon (PRKCE) and protein phosphatase slingshot homolog (SSH) 2, are involved in regulating actin dynamics and contractility^16, 18, 25, 26^. Myosin light chain kinase (MLCK) was also included because of its established role in regulating IOP and outflow facilities^27^. We found multiple genes listed above were regulated by miR-143/145 silencing in HTM cells (Fig. 5A,B). Specifically, ARPC-2, and -5 was significantly upregulated by either miR-143 or miR-145 silencing, while ARPC-3 was significantly upregulated by miR-143 only. PDGFRA and ABLIM-2 expression was upregulated by miR-143 and miR-145 silencing, respectively. That is consistent with the Targetscan prediction that PDGFRA is a miR-143 target gene^25^, and the PicTar prediction that ABLIM-2 is a miR-145 target gene^26^. Surprisingly, KLF5 was downregulated by either miR-143 or miR-145 silencing. This is in contrast to what occurs in vascular SMC cells, suggesting cell-type specific effect of miRNAs in target gene regulation^16^. MLCK expression was repressed by either miR-143 or miR-145 silencing. The expression of the other predicted miR-143 and/or miR-145 target genes in the list, including KLF4, SSH2, PRKCE, and ROCK1, was not significantly regulated by miR-143 and or miR-145 silencing. Together, our results show that multiple genes involved in actin dynamics and cell contractility, including ARPC-2, -3 and -5, PDGFRA, ABLIM-2 and MLCK, are regulated by miR-143/145 in HTM cells.

To further confirm whether miR-143 and miR-145 directly regulate their target genes, the activity of the 3′-untranslated region (UTR) of the target genes was analyzed by a reporter assay. ARPC-2, ARPC-3, ARPC-5 and PDGFRA 3′UTRs were cloned downstream of the coding region of luciferase in a cytomegalovirus (CMV)-driven luciferase vector, and tested for luciferase activity after co-transfection with miR-143 or miR-145 expression plasmids in COS-1 cells. PDGFRA and ARPC-5 UTRs contain canonical target sites for miR-143 and miR-145 respectively (Fig. 5C,D). Accordingly, overexpression of miR-143 repressed the PDGFRA 3′-UTR activity in a dose-dependent manner, while miR-145 failed to influence its activity (Fig. 5E). Similarly, miR-145 dose-dependently repressed the ARPC-5 3′-UTR activity (Fig. 5F). However, miR-143 also repressed the 3′-UTR activity of ARPC-2 and ARPC-5, suggesting the existence of non-canonical miR-143 targeting sites in the 3′-UTR of these two genes (Fig. 5F,G). Interestingly, neither miR-143 nor miR-145 significantly influenced the ARPC-3 3′-UTR activity, although they are capable of regulating its expression at mRNA level (Fig. 5H). These results suggest that miR-143 and miR-145 regulate genes involved in actin dynamics and cell contractility through both direct and indirect mechanisms.

## Discussion

The results of this study reveal an important role for miR-143 and miR-145 in regulating IOP in mice. The miR-143/145 miRNA cluster is expressed in the smooth muscle and the TM in the eye. Targeted deletion of miR-143 and miR-145 results in reduced IOP, consistent with an ~2-fold elevation in outflow facilities. These provide the first genetic evidence that miRNAs regulate IOP *in vivo*. Mechanistically, miR-143 and miR-145 are required for maintaining actin-cytoskeletal dynamics and contractility in HTM cells, possibly through regulating or directly targeting multiple genes involved in actin dynamics and contractility.

### Expression of miR-143/145 in the eye

Our data demonstrate that miR-143 and miR-145 are expressed in smooth muscle cells and TM cells in the eye. By qRT-PCR, the expression of miR-143 and miR-145 in human TM cells is less but comparable to the aortic SMC cells, but much higher than in choroidal endothelial cells and RPE cells. We have identified a 5.5 kb miR-143/145 upstream regulatory sequence that drives miR-143/145 expression in smooth muscle cells, pericytes, ciliary muscles and extraocular muscles. However, reporter gene activity was not detected in TM cells or corneal epithelial cells, suggesting that other regulatory regions may drive miR-143/145 expression in these cell types. Alternatively, the regulatory elements driving miR-143/145 in TM cells and corneal epithelial cells may not be conserved between human and mouse, or the reporter gene may not be sensitive enough to detect miR-143/145 expression in TM cells and corneal epithelial cells.

### Regulation of outflow facilities and IOP by miR-143/145

We provide genetic evidence that miR-143 and miR-145 regulate IOP *in vivo*. Although the gross ocular morphology is normal in *miR-143*/*145* dKO mice, there is an ~19% reduction in IOP compared to the WT littermates. When microbeads were used to obstruct the outflow facilities, the magnitude of IOP elevation was similar in both mice, indicating the aqueous humor production is largely normal in the dKO mice. Therefore, the IOP reduction in the dKO mice likely results from increased outflow facilities. This was confirmed by the *in vivo* experiments that showed a ~2-fold elevation in outflow facilities in the dKO mice. With regard to the miR-143/145 functional mechanism, we showed that miR-143 and miR-145 regulate actin-cytoskeletal dynamics and contractility in TM cells. In other reports, miR-143/145 has been shown to regulate actin dynamics and contractility in smooth muscle cells^16, 19^. The reduction in IOP in our model may result from the changes in actin-cytoskeletal dynamics and contractility in both TM and smooth muscle cells. Reduced smooth muscle contractility can cause a decrease in systolic blood pressure, which has shown to be associated with reduced IOP in humans^28^. It was reported that the systolic blood pressure in *miR-143*/*145* dKO mice is ~14 mmHg (14%) less compared to the wild-type littermates^29^. It is currently unclear regarding the contribution of blood pressure reduction, as well as the relative contribution of smooth muscle and TM cells, to the reduced IOP in our model.

### Regulation of actin dynamics and cell contractility by miR-143/145 in HTM cells

To study the mechanisms whereby miR-143 and miR-145 regulate outflow facilities, we investigated how these miRNAs regulate actin-cytoskeletal dynamics and contractility in TM cells. HTM cells show significantly reduced stress fiber length upon miR-143/145 silencing, suggesting a more relaxed or less stretched state in these cells. These results are in line with the previous results in vascular SMC^16^. Contractility of TMs and ciliary muscles has been shown to regulate aqueous humor outflow. Although the effect of miR-143/145 silencing on cell contraction was variable in primary HTM cells from different donors, our results suggest that these miRNAs, particularly miR-143, contribute to TM cell contractility. Similarly, miR-143 and miR-145 have been shown to regulate the contractility of vascular SMCs^19^. In line with observed phenotypes in HTM cells, multiple genes involved in actin dynamics and cell contractility are regulated by miR-143/145 as revealed by qRT-PCR. Among them, MLCK has established roles in regulating IOP and outflow facilities^27^. Inhibition of MLCK has been shown to lower IOP and increase outflow facilities. MLCK expression was repressed by either miR-143 or miR-145 silencing, although it is not a predicted miR-143/145 target. Expression of PDGFRA and ABLIM-2, which are miR-143 and miR-145 target genes respectively, was specifically upregulated by miR-143 and miR-145 respectfully. PDGFRA was further confirmed by 3′UTR luciferase assay to be a direct miR-143 target gene. PDGFRA has been shown to be involved in actin reorganization^30^. ABLIM-2 binds to F-actin and is localized to actin stress-fiber^31^. In addition, multiple members of the ARPC complex, including ARPC-2, -3 and -5, are upregulated by miR-143 or miR-145 silencing. Arp2/3 complex has been shown to play an essential role in generating branched actin filament networks^32^. The increased expression of ARPC5, and/or ARPC2/3 could potentially explain the reduced actin filament length upon miR-143/145 silencing. Among the ARPC genes, ARPC-2 and ARPC-5 were confirmed as direct miR-143 target genes, while ARPC-3 is probably regulated by miR-143/145 through indirect mechanisms. KLF5 is involved in vascular SMC proliferation and upregulated in miR-143/145 dKO aortas^16^. However, it was downregulated in HTM cells upon miR-143/145 silencing, which suggests cell-type specific regulation of miR-143/145 target genes. Future studies are needed to identify additional miR-143/145 regulated genes using an unbiased genome-wide approach.

### Therapeutic implications

Our findings that miR-143 and miR-145 regulate IOP have important therapeutic implications. Elevated IOP is the primary risk factor for blindness in glaucoma patients. Discovery of miRNAs as novel IOP regulators may provide new avenues for current glaucoma therapies. miR-143/145 silencing represses the expression of APRC, PDGFRA and MLCK genes. Among them, MLCK specific inhibitor ML-9 has demonstrated IOP-lowering effects in a rabbit model^27^. By simultaneously regulating multiple genes involved in actin dynamics, cell contractility and IOP regulation, miR-143/145 may provide distinct mechanisms to regulate IOP in glaucoma patients. Recently, delivery of miR-200c mimic by intracameral injection has been shown to decrease IOP by ~25%, illustrating the potential for miRNA therapeutics in glaucoma^15^. Future work should focus on examining the expression of miR-143/145 in glaucoma patients and testing the efficacy of miR-143/145 silencing in regulating IOP *in vivo* ^15^.

## Materials and Methods

### Animals and human samples

Animal studies were conducted in accordance with the ARVO statement for the Use of Animals in Ophthalmic and Vision Research and were approved by the Institutional Animal Care and Use Committees at the University of Texas Southwestern Medical Center, Tulane University and Duke University. All protocols involving human tissues were performed in accordance with the Tenets of the Declaration of Helsinki and approved by the Duke Health Institutional Review Board. *miR-143*/*145* dKO mice and the 5.5 kb miR-143/145 enhancer/promoter element-LacZ transgenic mice were generated as described^16^. Hematoxylin & Eosin staining and LacZ/Eosin staining were performed as described^16^. All experiments were performed in ~2 month mice with both sexes unless otherwise indicated. No sex-specific phenotypes have been observed. Outflow facilities were analyzed in ~16-month old mice (dKO mice: 4 females, WT mice: 2 females and 2 males; both eyes are used in the experiments).

### IOP measurement

IOP measurement was performed as described^33^. Each mouse was anesthetized, and topical application of 0.5% Proparacaine Hydrochloride (Alcon Laboratories, Fort Worth, TX) was placed on the corneal surface to maintain corneal moisture, which is vital in obtaining consistent measurements of IOP. Tono-pen tonometer (Mentor Corporation, USA) was calibrated before use as suggested in the manufacturer’s instructions. The calibration procedure was repeated until the digital display indicated having the highest reliability. All Tono-pen measurements were made without disposable latex cover over the tip. Every three times after the Tono-pen touched the cornea, its top was wiped clean by a 95% ethanol-soaked cotton-tipped applicator. 10 tonometer measurements were recorded and averaged for each eye.

### Induction of experimental glaucoma

Induction of experimental glaucoma was performed as described^34^. Mice were anesthetized with intraperitoneal injection of a ketamine (40 mg/kg) and Xylazine (10 mg/kg) cocktail. Each mouse was positioned on its side with the eye facing upward and head supported by a stack of sterile gauze. An intrastromal tunnel incision was made with a 30-gauge needle to puncture the mid peripheral cornea. A pulled glass micropipette with an inner diameter of 75 μm attached to a Hamilton syringe was inserted into the anterior chamber through the corneal incision and advanced to the center of the pupil. Care was taken not to touch the lens or the posterior surface of the cornea at any point. Polystyrene microbeads (FluoSpheres; Invitrogen, Carlsbad, CA; 15-μm diameter) were re-suspended at a concentration of 5.0 × 10^6^ beads/mL in PBS. 2 μL microbeads was injected through this preformed hole into the anterior chamber under a surgical microscope via the micropipette connected with a Hamilton syringe. After the procedure, all eyes were treated with a drop of 0.5% moxifloxacin. Only the right eyes were injected in all animals.

### Cell culture, antagomiR transfection, and phalloidin staining

Primary HTM cell lines were generated from donor eyes with no history of eye diseases as described previously^35^. The cells were cultured in DMEM (low glucose), with 10% FBS, Penicillin/Streptomycin, and non-essential amino acids. The purity of the cells was verified by observing the morphology and the growth rate of the cells. The morphology of the cultures used in this study was consistent with that of typical TM cells with no signs of fibroblast contamination. Human Aortic Smooth Muscle Cells (HASMC) were purchased from Lonza and cultured in SmGM-2 medium (Lonza). HCEC cells were kindly provided by Dr. Ashwath Jayagapol from Vanderbilt University and grown in EGM2 media (Lonza). ARPE-19 (ATCC) cells were grown in DMEM/F12 (HyClone) media with 10% FBS. miR-143, miR-145 and control antagomiRs were ordered IDT. The sequences include: 2′-O-methyl anti-miR-143: 5′-mGmAmG mCmUA CAG UGC UUC AmUmC mUmCmA3′; 2′-O-methyl anti-miR-145: 5′-mAmGmG mGmAU UCC UGG GAA AAC mUmGmG mAmC-3′; 2′-*O*-me-scrambled miR: 5′-mAmAmAmAmCCUUUUGACCGAGCmGmUmGmUmU-3′. miR-143/145 AntagomiR transfection and phalloidin staining were performed as described^36^. Briefly, antagomiR transfection was performed in HTM cells using Lipofectamine® RNAiMAX transfection reagent (ThermoFisher Scientific) at a concentration of 50 nM for antagomiR. Cells were cultured for additional 72 hours unless otherwise indicated before processing for different assays. For phalloidin staining, HTM cells were fixed with 4% paraformaldehyde and stained with 50 mg/ml fluorescein isothiocyanatelabeled phalloidin (Sigma) at room temperature for 1 hour. Quantification of F-actins amount was performed using the Image J.

### Quantitative (q) RT-PCR for mRNAs and miRNAs

qRT-PCR was performed according to the MIQE guidelines. Total RNA was isolated with TRIZOL reagent (Invitrogen) according to the manufacturer’s protocol. To enrich both mRNA and miRNA in the samples, 1 volume of isopropanol (instead of 0.5 volume listed in the manual) was added to the samples, and the samples were incubated at −80 °C for 15 minutes before RNA precipitation. qRT-PCR was performed using iScript™ cDNA Synthesis system (Bio-Rad) and sybgreen qPCR system from invitrogen, and miRNA qRT-PCR was performed using a microRNA cDNA synthesis kit and microRNA Quantification System (Quanta Biosciences). qPCR was performed using CFX96™ real-time PCR system (Bio-Rad) using the following conditions: 10 minutes at 95 °C for denaturation, followed by 45 cycles of 15 seconds at 94 °C for denaturation and 60 seconds at 94 °C for annealing and extension. Primer melting curve analysis was performed after PCR reaction as below: 31 seconds at 65 °C, followed by 60 cycles of the heating procedures (5 seconds at 65 °C, with 0.5 °C increase/cycle and a ramp of 0.5 °C/second). Data analysis was performed using Bio-Rad CFX Manager software using Cyclophilin (for mRNA PCR) or RNU6 (for miRNA PCR) as normalization controls, and control transfection as sample controls. Additional control primers, including β-actin for mRNA and SNORD44 for miRNA, were also routinely used to check for consistency between different controls. miR-143 and miR-145 PCR primers were purchased from Quanta. Other primers were synthesized by IDT, including: hARPC2: 5′-ggaaggagagaacagggcagt-3′ and 5′- cctttccaatgaccacatcgt-3′; hARPC3: 5′-acgcaatttatgccaaacctg-3′ and 5′-gtccaccacttgctgggttta-3′; hARPC5: 5′-gttcgtggacgaagaagatgg-3′ and 5′-tcttcagagctgcctgtaggg-3′; hKLF5: 5′-aggagtccacccgaaacct-3′ and 5′-tcatgctcagcacccttgta-3′; hMLCK: 5′-ccgaggttgtctggttcaaag-3′ and 5′-cagttcccgtcctcatcgtag-3′; hPDGFRA: 5′-tgtgggacattcattgcgga-3′ and 5′-taggctcagccctgtgagaa-3′; hABLIM2: 5′-gatgaccggtcctacaagcag-3′ and 5′-ggcagctactggtacccacac-3′; hPRKCE: 5′-gaccaaggaccgcctctttt-3′ and 5′-ccatgctggtggaggaacat-3′; hSSH2: 5-gcctcgagctctcatttgga-3′ and 5′-ccactgtctgcctcattgga-3′; hROCK-1: 5′-ccaccatctggttttgttcgt-3′ and 5′-gattccacagggcactcagtc-3′; hKLF4: 5′-ccttcctgcccgatcagatg-3′ and 5′-cgtcttcccctctttggctt-3′.

### Plasmid construction and luciferase assays

PDGFRA, ARPC-2, ARPC-3 and ARPC-5 3′ untranslated regions (UTRs) were PCR amplified from cDNA from HTM cells and cloned into the pMIR-REPORT vector (Ambion) after digestion with Sac I and Hind III enzymes. The positive plasmids were confirmed by sequencing (IDT). miR-143 and miR-145 expression plasmids were described in ref. 16. miR-143, or miR-145, expression plasmid was co-transfected with the reporter plasmids in COS1 cells and reporter assays were performed as described^36^. Sequences for PDGFRA UTR cloning are: 5′-atcgGAGCTCcgaggggttccttccacttc-3′ and 5′-atcgAAGCTTacgatggattggggaaccta-3′. Sequences for ARPC-2 UTR cloning are: 5′-atcgGAGCTC CTTGGGAATAAGAGGAGGAAG-3′ and 5′-atcgAAGCTTGCATTATCCTGCAACTCATTAC-3′. Sequences for ARPC-3 UTR cloning are: 5′-atcgGAGCTC GCAGCCACCGTCTCCAG-3′ and 5′-atcgAAGCTT ACTTATTCTTATTAAGCGCCAGC-3′. Sequences for ARPC-5 UTR cloning are: 5′-atcgGAGCTCtatctggctcgggagtggga-3′ and 5′-atcgAAGCTTtccctcttggtcaggtggtt -3′.

### Analysis of outflow facilities in living mouse eyes

Outflow facilities was evaluated in anesthetized mice as previously described^37^. Briefly, two glass micro-needles filled with PBS were inserted into both anterior chambers through the corneas with the aid of micromanipulators. Each micro-needle was connected with a vertical fluid column and pressure transducer (Honeywell model 140PC; Honeywell Sensing and Control, Freeport, IL) through pressure tubing and four-way stopcocks. The pressure transducer was linked to a data acquisition system (ML870/P PowerLab; AD Instruments, Colorado Springs, CO). The pressures were zeroed when the needle tips were immerged into tear films before they were placed into eyes. After the needles were inserted into eyes, the pressures were raised sequentially to initial 15, 25, and 35 mmHg at 20 min intervals. The outflow was calculated by monitoring the slight decline in pressure over time resulting from fluid exiting the system from the vertical fluid column at each pressure level. Outflow facilities was determined by the slope from linear regression curve of flow versus pressures.

### Contractility assay

Cell contractility assay was performed as described^15^. Collagen gels were prepared in 24 well plates from rat tail collagen type 1 (1.5 mg/ml, BD Biosciences, Bedford, MA) following manufacturer’s instructions. After 24-hour transfection with miR-143 or miR-145 antagomiRs, HTM cells were embedded in the collagen preparation before pouring, and polymerized at 37 °C, 5% CO_2_ for 30 minutes. Complete media was added after polymerization and gels were incubated for another 48 hours. Cells were changed to serum free medium, and after 2 hours, the gels were detached from the walls and photographed 24 hours later. The gel area was calculated using Image J software and transformed from arbitrary units to mm^2^. The effect of these antagomiRs on the levels of cell contraction was calculated as the difference in gel area between scramble and miR-143 or miR-145 anti-miRs.

### Statistics

All the data were analyzed using Graphpad prism 7 software. The data in the text was presented as mean +/− SEM, with the number of samples indicated. Student’s T-tests were used to determine statistical significance between groups. P-values of less than 0.05 were considered to be statistically significant.

## Electronic supplementary material

Supplemental Fig
