# Supplementary material for: Regulation of intraocular pressure by microRNA cluster miR-143/145
Source: Sci Rep. 2017 Apr 19;7:915. doi: 10.1038/s41598-017-01003-z (PMC5430458; doi:10.1038/s41598-017-01003-z)

Regulation of intraocular pressure by microRNA cluster miR-143/145

Xinyu Li[1, 5], Fangkun Zhao[2, 3], Mei xin[6], Guorong Li[8], Coralia Luna[8], Guigang Li[1], Qinbo Zhou[3], Yuguang He[5], Bo Yu[3], Eric Olson[7], Pedro Gonzalez[8] and Shusheng Wang[3, 4*]

Supplemental Figure 1

(A) Visualization of smooth muscle cells in the ciliary body in wild-type mice by α-SMC staining;

(B) Pseudo-coloring of the bright field picture in (A);

(C) Visualization of smooth muscle cells in the choroid in wild-type mice by α-SMC staining;

(D) Pseudo-coloring of the bright field picture in (C);

(E) Visualization of pericytes in the retina in wild-type mice by NG-2 staining;

(F) Pseudo-coloring of the bright field picture in (E). Scale equals 40μm.

Supplemental Figure 2

(A) Histological H&E staining showing normal extraocular muscle structure in *miR-143/145* dKO mice. Scale bar=50μm;

(B) Normal extraocular muscle structure in *miR-143/145* dKO mice as shown by transmission electron microscopy analysis. Scale bar=2μm.

Supplemental Figure 1

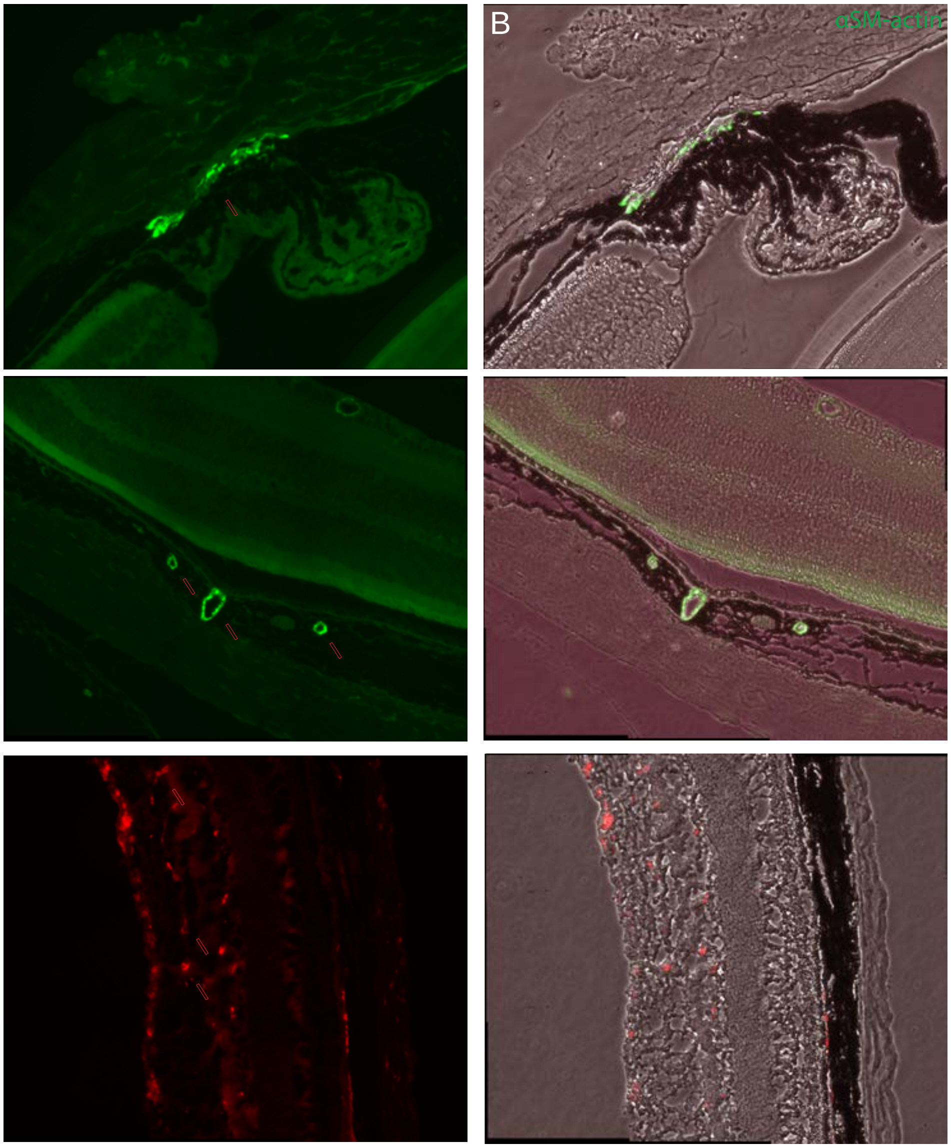

Supplemental Fig. 2

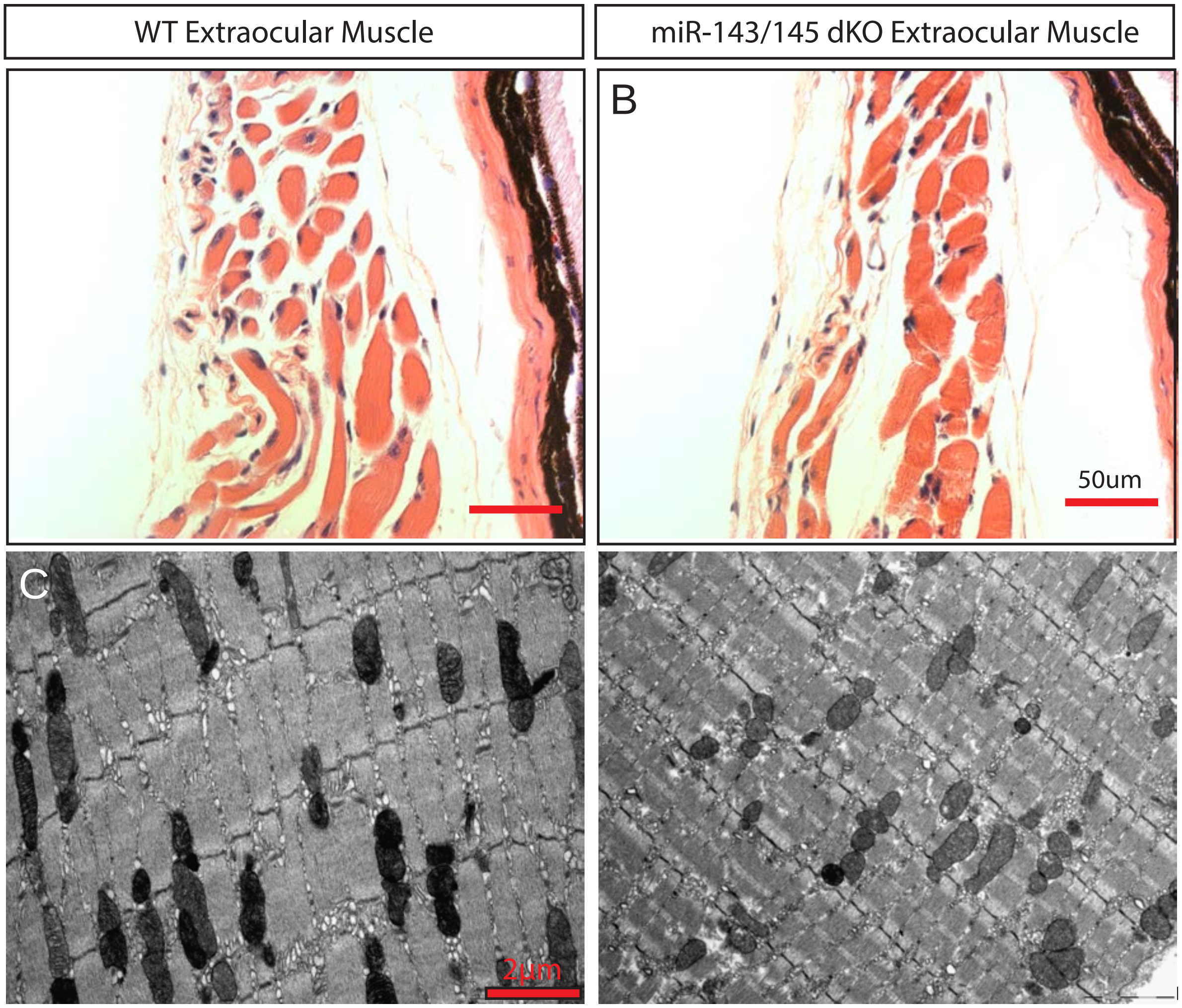

Supplement: Supplementary file 1 — Supplemental Fig [file 41598_2017_1003_MOESM1_ESM.pdf]
